# Profiling vaccine hesitancy in nursing to tailor public healthcare policies: A cross‐sectional international study

**DOI:** 10.1111/jnu.13016

**Published:** 2024-08-14

**Authors:** Jemma McCready, Goran Erfani, Dania Comparcini, Giancarlo Cicolini, Kristina Mikkonen, Jeremia Keisala, Marco Tomietto, the Sigma IMPACT Research team, Charlotte Gordon, John Unsworth, Michelle Croston, Valentina Simonetti, Bethany Nichol

**Affiliations:** ^1^ Department of Social Work, Education and Community Wellbeing, Faculty of Health and Life Sciences Northumbria University Newcastle upon Tyne UK; ^2^ Department of Nursing, Midwifery and Health, Faculty of Health and Life Sciences Northumbria University Newcastle upon Tyne UK; ^3^ Section of Nursing, Department of Precision and Regenerative Medicine and Ionian Area University of Bari “Aldo Moro” Bari Italy; ^4^ Department of Innovative Technologies in Medicine and Dentistry “Gabriele D'Annunzio” University of Chieti Chieti Italy; ^5^ Medical Research Center Oulu Oulu University Hospital and University of Oulu Oulu Finland; ^6^ Research Unit of Health Science and Technology Faculty of Medicine University of Oulu Finland

**Keywords:** COVID‐19, influenza, nursing community, profiling, vaccine hesitancy

## Abstract

**Introduction:**

Vaccine hesitancy is a complex issue of global concern. As nurses play a vital role in delivering patient care and shaping public opinions on vaccines, interventions to address vaccine hesitancy in nursing are imperative. As such, identifying profiles of characteristics and attitudes contributing to hesitancy may help identify specific areas of focus to target tailored global vaccination uptake campaigns. The purpose of this study was to profile the characteristics and attitudes contributing to hesitancy toward COVID‐19 and Influenza vaccines in the nursing community.

**Design:**

This multisite, cross‐sectional study recruited 1967 registered nurses and 1230 nursing students from the United Kingdom, Finland, and Italy between March and September 2023.

**Methods:**

Data collection involved an online survey adopting the Vaccination Attitudes Examination (VAX) Scale, the Bergen Social Media Addiction Scale, and questions pertaining to sociodemographic and occupational characteristics. A k‐means cluster analysis was used to identify various clusters of hesitancy based on the VAX Scale. One‐way ANOVA and chi‐square tests were used to identify significant differences in sociodemographic characteristics, occupational factors, vaccination attitudes, and social media usage between the clusters.

**Results:**

Three distinct clusters were identified. Profile A showed high vaccine confidence, profile B displayed slight hesitancy, and profile C reported high levels of hesitancy. In profile C, higher levels of vaccine hesitancy were identified in younger, less experienced nurses with lower educational attainment. While older nurses with higher educational attainment, who were in senior roles, were more vaccine‐confident and had a consistent history of accepting the Influenza and COVID‐19 vaccinations (profile A). The study found Italian nurses highly hesitant (profile C), British nurses highly confident (profile A), and Finnish nurses evenly distributed between confident, slightly hesitant, and highly hesitant (profiles A, B, and C, respectively). In addition, more frequent usage of Instagram and TikTok was associated with vaccine hesitancy (profiles B and C), and LinkedIn and X were more common among vaccine‐confident individuals (profile A).

**Conclusions:**

This study has identified specific sociodemographic and occupational factors that are related to vaccine hesitancy in an international sample of nurses. Additionally, attitudes contributing to hesitancy were identified, with worries about unforeseen future effects of the vaccine being identified as a critical attitude that may undermine confidence and increase hesitancy in nursing. This study also sheds light on the influence that social media platforms have on vaccine hesitancy and, as such, indicates which platforms are effective to disseminate vaccination campaigns to global nursing communities.

**Clinical Relevance:**

Global vaccination campaigns should focus on specific profiles and clusters to promote vaccination in the international nursing community. Empowering nurses early in their careers will help to instill positive vaccination behaviors, ensuring a sustained uptake of vaccinations throughout the individual's career and beyond, with an impact on promoting vaccination at the public health level as well.

## INTRODUCTION

Vaccine hesitancy is defined by the World Health Organization (WHO, 2015) as the delay in accepting or rejecting vaccines despite availability. Moreover, it is a worldwide concern that poses risks to the health infrastructure, economy, and safety of countries (WHO, 2018). The consequences of insufficient vaccination coverage became evident during the COVID‐19 pandemic, where vaccination was the most important defense strategy to reduce the transmission of SARS‐CoV‐2 and to prevent hospitalizations and fatalities (WHO, 2020). However, the need for implementing vaccination uptake has been a public health priority since many years, for example, regarding the seasonal influenza vaccination campaigns (ECDC, 2018). As such, global efforts were made to ensure the distribution and uptake of vaccinations. Although initial vaccine rollouts garnered public support—with around 710 million doses being accepted globally in the first 4 months (Mathieu et al., 2021)—there was, and there remains, around 20% of the global population who remain hesitant to accept vaccination (Lazarus et al., 2023).

Research into groups with high levels of hesitancy identified that healthcare workers (HCWs), specifically nurses, were hesitant to accept both COVID‐19 and influenza vaccines (Khubchandani et al., 2022). For example, the vaccination uptake among HCWs for seasonal influenza vaccine is <40% in European countries (ECDC, 2018). Vaccine hesitancy among healthcare workers is a key element for a safe and effective healthcare provision and closely linked to occupational risk of infection, the risks to immunocompromised and vulnerable patients, and the challenges associated with workforce availability (Maltezou et al., 2022). As such, suboptimal uptake of a vaccine prompted some governments to introduce mandatory vaccination policies for HCWs (Maneze et al., 2023), and especially during the pandemic, with refusal resulting in redeployment, reduced hours, and unemployment (Tobin, 2021). Within Europe, countries such as Italy, Finland, France, and Germany implemented vaccination mandates, while other countries, such as the United Kingdom, revoked their mandates before rollout (Department of Health and Social Care, 2018; Karlsson et al., 2023). Such a strategy was criticized for impinging and violating human rights, particularly personal autonomy. Critics argue that these measures could further contribute to hesitancy by increasing fear and mistrust of government and health authorities (Drew, 2019), exacerbating workforce shortages, and fostering division within the healthcare sector (Royal College of Nursing, 2022). While the immediate threat from the COVID‐19 pandemic may have diminished, it is crucial to enhance adherence to vaccination and preparedness for mitigating responses to potential future outbreaks and yearly seasonal influenza recurrence. This knowledge will enable a more informed and adept approach to addressing vaccine hesitancy in healthcare workforces, specifically for nurses.

Nurses are the backbone of healthcare institutions and, as such, play a vital role in delivering care to patients. The frontline nature of the job makes nurses more vulnerable to contracting COVID‐19 and influenza and a vector of transmission to other clinically vulnerable patients, colleagues, and the wider population (Asad et al., 2020). Moreover, nurses are a trusted and reliable source of information for the general population, with research indicating that recommendations from nurses improve vaccination uptake within the wider community (Tomietto et al., 2022). Therefore, they are an influential figure to include in public health strategies, making it imperative that their reasons for vaccine hesitancy are addressed. A recent systematic review highlights that vaccine hesitancy among nurses is a complex issue associated with various factors. These include individual characteristics such as sociodemographic (e.g., female gender, lower educational attainment), occupational responsibilities (e.g., less work experience, being a trainee, less perceived risk of infection), vaccination history (e.g., not up to date with the Influenza vaccine), and health status (e.g., presence of chronic diseases) (McCready et al., 2023). Additionally, certain attitudes and beliefs contribute to increased hesitancy, such as concerns about vaccine efficacy, side effects, safety/speedy approvals, and mistrust in governmental and healthcare agencies (Khubchandani et al., 2022). Moreover, exposure to misinformation or disinformation on social media sites also emerged as a significant factor influencing hesitancy among nurses (Khubchandani et al., 2022).

Alongside nurses, it is also crucial to consider nursing students' vaccine hesitancy, as they spend 50% of their time in healthcare settings and clinical placement (EUR‐Lex, 2019) and are at risk of both contracting communicable diseases and spreading them to patients (Asad et al., 2020). Furthermore, intention to receive the seasonal influenza vaccine ranges between 15% and 33% in this population (Salem et al., 2019), and it is 44% when considering the COVID‐19 vaccine (Patelarou et al., 2021).

Although several studies have identified specific characteristics and attitudes associated with vaccine hesitancy in nursing, very few studies have applied a person‐centered approach that clusters nurses and nursing students into specific profiles based on different levels of vaccine hesitancy. The World Health Organization (2019) has stated as public health priority the need for further research to understand the perceptions, motivators, and barriers to vaccine acceptance among healthcare workers. This study provides new insights to vaccine hesitancy by identifying latent profiles of specific characteristics contributing to vaccine hesitancy in nursing, by considering both nurses and nursing students. Moreover, in order to develop a comprehensive global strategy, this study involved participants from the United Kingdom, Finland, and Italy to assess whether there are common factors influencing vaccination uptake from a global health perspective and independently from the social, cultural, and political factors. Insights from this study will help identify specific profiles of nurses and nursing students and pinpoint areas of focus for a global vaccination campaign in public health. Such a targeted approach is useful to identify the most hesitant profiles and to improve vaccination uptake with an impact on patient safety and global health.

### Aim

This study aims to profile the characteristics and attitudes contributing to vaccine hesitancy toward the COVID‐19 and influenza vaccines among nurses and nursing students.

## MATERIALS AND METHODS

### Design

A multisite, cross‐sectional study design was utilized to gather sociodemographic characteristics and attitudes toward COVID‐19 and influenza vaccines to determine levels of vaccine hesitancy in nurses and nursing students from the United Kingdom, Italy, and Finland. The Strengthening the Reporting of Observational Studies in Epidemiology (STROBE; Vandenbroucke et al., 2007) guidelines were used to guide the reporting of this study.

### Participants and sample size

Registered nurses and nursing students were recruited through both formal and informal networks using social media platforms, academic networks, and professional associations. To be eligible for the study, participants had to be a registered nurse, currently working within healthcare settings, or nursing students.

For confirmatory factor analysis (CFA), it is recommended to have a participant‐to‐item ratio ranging from 10:1 to 20:1 (Kline, 2015). Consequently, the necessary sample size fell between 120 and 240 participants.

For K‐cluster analysis, calculating a predetermined sample size for comparison across clusters was not possible a priori, as this is an unsupervised machine‐learning approach. Thus, the sample size estimation was computed by considering a range of clusters from 3 to 5. In detail, by considering three clusters, an alpha error of 0.05, a power of 0.95, and an effect size of 0.25, a total sample size of 252 participants per country was considered adequate. By considering five clusters, using the same parameters, a sample size of 305 participants per country was recommended.

Overall, 1967 nurses and 1230 nursing students participated in this study. In detail, 312 nurses were from the United Kingdom, 302 from Italy, and 1353 from Finland. Among nursing students, 310 were from the United Kingdom, 340 were from Italy, and 580 were from Finland. Given the large Finnish sample and to balance the international comparison, 310 nurses and 310 nursing students were randomly extracted from the Finnish sample via the random extraction function in SPSS v28 (IBM Corp., 2021).

### Data collection

Data were collected through an online survey built on the survey platform JISC between March and September 2023. A convenience sampling approach was adopted, combined with a snowballing approach.

### Data analysis

The data were analyzed using SPSS v28 (IBM Corp., 2021). The confirmatory factor analysis was carried out using Stata v13 (StataCorp., 2013). Multivariate outliers were identified by computing the Mahalanobis distances. Identifying and removing multivariate outliers was essential to meet the assumptions for computing multivariate statistics (Kline, 2015). Furthermore, multivariate outliers affect the integrity of K‐means cluster analysis, leading to suboptimal clustering outcomes (Ikotun et al., 2023). To test multivariate normality, Mardia's kurtosis was computed, where a value lower than the critical threshold of v*(v + 2) (v being the number of items) indicates multivariate normality (Mikkonen et al., 2022; Tabachnick et al., 2013). Where applicable, the Little's MCAR test was calculated to test if missing data were Missing Completely at Random (MCAR) (Mikkonen et al., 2022).

K‐means cluster analysis was used to identify the clusters of hesitancy based on the VAX scale's factors. The suitable number of clusters was detected through a two‐step cluster analysis using silhouette measures of cohesion by adopting the Euclidean method.

One‐way ANOVA and chi‐square tests were employed to ascertain the statistical significance of differences among clusters. A *p* < 0.05 was deemed indicative of adequate statistical significance. Categorical data were represented using frequencies and percentages in the statistical analysis. Meanwhile, continuous variables, specifically the VAX scale scores, were presented as mean values and standard deviations (SD).

#### Preliminary analysis: Multivariate outliers, normality, and missing data

In this study, the Mardia's kurtosis value, without eliminating multivariate outliers, was 822.87, and the critical value was 624. By deleting the multivariate outliers, Mardia's kurtosis reduced to 549.71, confirming multivariate normality.

Over a total sample of 1884 participants, 128 multivariate outliers were identified and deleted for data analysis, resulting in a final sample of 1756 participants. Specifically, 293 nurses were from the United Kingdom, 271 from Italy, and 295 from Finland. Among nursing students, 281 were from the United Kingdom, 326 from Italy, and 290 from Finland. As the VAX scale for both influenza and COVID‐19 was mandatory, there was no need for missing data analysis.

The Bergen Social Media Addiction Scale was not mandatory; therefore, the Little's Missing Completely at Random (MCAR) test was calculated to ensure the missing data were MCAR. The test showed a *p*‐value of 0.89 (*χ* ^2^ = 14.91, df = 23), confirming the data were MCAR.

### Measures

The Vaccination Attitudes Examination (VAX) Scale was employed to measure COVID‐19 and influenza vaccine hesitancy. The scale included 12 items rated on a Likert scale ranging from 1 (totally disagree) to 7 (totally agree) (Martin & Petrie, 2017). The 12 items are grouped into four factors: “mistrust of vaccine benefit” (three items—reversed), “worries about unforeseen future effects” (three items), “concerns about commercial profiteering” (three items), and “preference for natural immunity” (three items) (Martin & Petrie, 2017). Lower scores indicate a more favorable attitude toward vaccines.

The Bergen Social Media Addiction Scale (BSMAS; Andreassen et al., 2017) was utilized to evaluate Social Media Addiction (SMA). The scale includes six items that assessed social media usage using a 5‐point Likert scale, with the anchor representing “very rarely” (1) to “very often” (5). Higher scores are indicative of a more addictive attitude toward using social media.

Additionally, data on sociodemographic characteristics (i.e., age, gender, country of residence, and educational attainment), occupational factors (i.e., occupational role, work experience, professional experience, and occupational setting), health‐related factors (i.e., exposure to COVID‐19 infection and vaccination history), and social media usage (i.e., frequency of usage of various social media platforms rated from never (1) to always (4)) were collected to characterize the sample and to facilitate comparisons of characteristics between the clusters.

### Validity, reliability, and rigor

#### Translation procedure and content validity

A panel of researchers evaluated the scale and provided linguistic and cultural adaptation to the different national contexts. A forward and backward translation was performed. Each national panel preliminarily translated the English version into their language and achieved agreement on the national translation. Each national version was blindly back‐translated into English by a native English speaker. Finally, the original English version and the English back‐translated version were blindly compared by another researcher, fluent in English and familiar with the topic. An independent researcher stated the content equivalence of the two versions and, therefore, the content validity of the translated versions (Maneesriwongul & Dixon, 2004).

#### Validity and reliability

Cronbach's αwas computed for each factor within the VAX scale, evaluating the reliability of both the influenza and COVID‐19 scales. Values >0.70 are considered adequate (DeVellis & Thorpe, 2021).

CFA was conducted and fit indices were calculated. Acceptable fit indices are indicated by an RMSEA (root mean square error of approximation) and SRMR (standardized root mean residual) of <0.08, and for CFI (comparative fit index) and TLI (Tucker–Lewis index), values >0.90 are considered adequate (Kline, 2015).

In this study, the reliability of the VAX scale for influenza showed a Cronbach's *α*of 0.92 for the overall scale, with values ranging between 0.80 and 0.90 across the four factors. For the VAX scale associated with the COVID‐19 vaccine, the overall Cronbach's *α*was 0.94, with values spanning from 0.85 to 0.94. The Cronbach's *α*for the BSMAS was 0.84.

The fit indices for the VAX scale concerning influenza displayed the following results: RMSEA = 0.073 (90% CI = 0.067–0.079), SRMR = 0.037, TLI = 0.957, and CFI = 0.968. Meanwhile, the fit indices for the VAX scale related to the COVID‐19 vaccine were as follows: RMSEA = 0.070 (90% CI = 0.064–0.076), SRMR = 0.032, TLI = 0.972, and CFI = 0.980. For the BSMAS, the observed fit indices were as follows: RMSEA = 0.082 (90% CI = 0.065–0.099), TLI = 0.955, and CFI = 0.979 (SRMR not calculated due to missing values). In this study, all scales verified reliability and validity.

### Ethical considerations

The data collection and analysis procedures were structured to ensure data confidentiality and compliance with national and European laws, encompassing the General Data Protection Regulations (Cornock, 2018) and the UK Data Protection Act (2018). To uphold data security, electronic data were securely stored in a protected folder accessible solely to the principal investigator and the designated research team. Participants were presented with a comprehensive disclaimer outlining study details and information regarding data handling on the initial survey page. By voluntarily submitting the survey, participants explicitly provided their informed consent to engage in the study. Ethical approval for this study was obtained from Northumbria University (ref: 2948, date: 27/02/23), and consent was obtained in accordance with the Declaration of Helsinki.

## RESULTS

### Participant characteristics

The respondents had an average age of 34.83 years (SD = 13.37, median = 31, min = 18, max = 70), and most respondents were female (83.6%). The number of participants in the final sample across the United Kingdom, Finland, and Italy were 574 (32.7%), 585 (33.3), and 597 (34%), respectively. The sample included 859 (48.9%) registered nurses and 897 (51.1%) nursing students. The nurse respondents reported an average work experience of 9.77 years (SD = 9.38, median = 6, min = 0, max = 46) in their current area of clinical practice. The average work experience in the nursing profession, measured in years post‐qualification, was 18.65 years (SD = 12.35, median = 17, min = 0, max = 50). Nurses reported their highest academic qualifications as Diploma/Advanced Diploma 234 (27.2%), BSc/BSc (Hons) 417 (48.5%), and MSc/PhD 208 (24.2%). The nursing students were mainly undergraduate students (*n* = 869, 96.9%) attending the first year of study (*n* = 300, 34.5%), the second year (*n* = 334, 38.4%), and the third year (*n* = 235, 27.1%). The remaining were post‐graduate nursing students (*n* = 28, 3.1%).

### Cluster analysis

The K‐means clustering technique with log‐likelihood as distance measure successfully identified a maximum of three distinct profiles from the dataset. These profiles were labeled as profile A (*n* = 678), profile B (*n* = 732), and profile C (*n* = 346) (Table 1). The three‐cluster solution had a silhouette measure of cohesion and separation of 0.4, indicating a “fair” quality. Moreover, the one‐way ANOVA test, followed by the Bonferroni post hoc test, provided further support for the robustness and validity of the three‐cluster solution. This was evident as all pairwise comparisons between profiles for each of the eight VAX factors demonstrated a highly significant level of *p* < 0.001. However, when considering four or five clusters, no significant differences were observed between the detected clusters for all factors. Consequently, the three‐cluster solution proposed in this study was validated.

**TABLE 1:** Sample profiles.

VAX factors	Profile A (*n* = 678) Mean (SD)	Profile B (*n* = 732) Mean (SD)	Profile C (*n* = 346) Mean (SD)	*F* ^a^	*p*‐value
COVID‐19					
Mistrust of vaccine benefit	1.90 (0.83)	2.77 (0.96)	4.85 (1.45)	941.30	**<0.001**
Worries about unforeseen future effects	3.42 (1.09)	4.94 (0.88)	5.81 (0.95)	800.82	**<0.001**
Concerns about commercial profiteering	1.46 (0.56)	2.69 (0.92)	4.89 (1.13)	1846.75	**<0.001**
Preference for natural immunity	1.80 (0.79)	3.37 (0.97)	4.90 (1.09)	1332.98	**<0.001**
Overall scale	2.14 (0.49)	3.44 (0.47)	5.11 (0.82)	3281.59	**<0.001**
Influenza					
Mistrust of vaccine benefit	1.72 (0.76)	2.43 (0.81)	4.07 (1.36)	734.24	**<0.001**
Worries about unforeseen future effects	3.29 (1.04)	4.78 (0.88)	5.58 (0.92)	784.62	**<0.001**
Concerns about commercial profiteering	1.46 (0.55)	2.56 (0.85)	4.39 (1.08)	1525.93	**<0.001**
Preference for natural immunity	1.87 (0.78)	3.31 (0.90)	4.61 (1.05)	1167.14	**<0.001**
Overall scale	2.09 (0.44)	3.27 (0.45)	4.66 (0.71)	3045.17	**<0.001**

*Note*: The mean difference is statistically significant at *p* < 0.001 or higher (highlighted in bold). The vaccination hesitancy score was based on a 7‐point Likert scale (scores 1–7).

^a^ One‐way ANOVA *F* test, including multiple pairwise comparisons conducted with Bonferroni correction; each comparison demonstrated a *p* < 0.001 or higher.

### Vaccine hesitancy profiles

Participants clustered in profile A exhibited the lowest mean scores for each VAX factor compared to participants in the other two profiles. As a result, profiles A, B, and C can be classified as having low, average, and high vaccine hesitancy, respectively. The overall mean scores for COVID‐19 vaccine hesitancy were 2.14 (SD = 0.49) for profile A, 3.44 (SD = 0.47) for profile B, and 5.11 (SD = 0.82) for profile C. For influenza vaccine hesitancy, the overall mean scores were 2.09 (SD = 0.44) for profile A, 3.44 (SD = 0.47) for profile B, and 4.66 (SD = 0.71) for profile C. Overall, participants in all three clusters reported a greater vaccine hesitancy for COVID‐19 VAX factors and overall than for influenza (Table 1).

In terms of attitudes toward vaccination, participants in all three profiles identified “worries about unforeseen future effects” as the primary attitude that prompted high levels of hesitancy. For the COVID‐19 vaccination, the mean scores for this item were 3.42 (SD = 1.09) for profile A, 4.94 (SD = 0.88) for profile B, and 5.81 (SD = 0.95) for profile C. For the influenza vaccination, the mean scores were 3.29 (SD = 1.04) for profile A, 4.78 (SD = 0.88) for profile B, and 5.58 (SD = 0.92) for profile C. Participants in profile A were least hesitant due to “*concerns about commercial profiteering”* of COVID‐19, with a mean score of 1.46 (SD = 0.56). Participants in profile B scored their “*mistrust of vaccine benefits”* for influenza the lowest, with a mean score of 2.43 (SD = 0.81). Participants in profile C also reported the lowest score for “*mistrust of vaccine benefits”* for the influenza vaccine, with a mean score of 4.07 (SD = 1.36), indicating their most positive attitudes toward vaccination (Table 1).

### Profiling participants' characteristics and comparison

In the overall sample, a statistically significant age difference was observed, with the participants in profile A (mean = 36.75, SD = 14.25) being approximately 3 years older than those in profile C (mean = 33.50, SD = 11.92). Profile A was most commonly represented by nurses (56.5%), while profile C was predominantly represented by nursing students (57.5%). The differences between countries were also statistically significant (*χ* ^2^ = 45.974, *p* < 0.001). Most participants clustered in profile A (38%) were British, while most reported participants were Italian in profiles B (41.9%) and C (33.5%). Finnish participants reported the lowest frequency in profile C (31.5%).

Regarding the use of social media applications, participants in profile A (mean = 2.86 SD = 1.13) reported a lower level of Instagram usage compared to participants in profile B with a mean score of 3.07 (SD = 1.05). In addition, participants in profile A reported a lower level of using TikTok, with a mean score of 1.99 (SD = 1.22), than participants in profile C (mean = 2.24, SD = 1.23). A similar statistically significant pattern was observed for comparing using Snapchat among participants clustered in profile A (mean = 1.58, SD = 1.05) and profile C (mean = 1.85, SD = 1.17). Conversely, participants in profile A reported a higher level of using Reddit, with a mean score of 1.25 (SD = 0.66), than participants in profile C (mean = 1.09, SD = 0.33). Participants in profile A also reported the highest level of using LinkedIn and Twitter/X, with mean scores of 1.40 (SD = 0.71) and 1.80 (SD = 1.07), respectively. In terms of using Pinterest, participants in profile B reported a higher level of usage, with a mean score of 1.58 (SD = 0.70), than participants in profile C (mean = 1.45, SD = 0.70).

Most participants in profiles A (77%) and B (59%) reported getting vaccinated for influenza on a yearly basis. In contrast, the majority of participants in profile C reported that they had not received an influenza vaccine either at all or not on a yearly basis (65%). This difference in influenza vaccination history between the profiles was statistically significant (*χ* ^2^ = 198.874, *p* < 0.001). Regarding the COVID‐19 vaccine, the majority of participants in each profile had received a COVID‐19 inoculation (profile A = 97%, profile B = 94%, and profile C = 75%). However, profile C contained a significantly greater number of unvaccinated individuals than identified in profiles A and B (25%, 3%, and 6%, respectively; *χ* ^2^ = 157.528, *p* < 0.001).

When specifically focusing on nurses, the frequency of senior roles (nurse leader and educators) reduced from 35.4% in profile A to 28.8% in profile B and 23.3% in profile C. Nurses in profile A reported the most years of work experience post qualification, with a mean of 20.31 years (SD = 12.73), while nurses in profile C reported the least extensive work experience, with a mean of 16.21 years (SD = 12.45). Regarding the highest academic award, nurses with higher degrees were most often represented in profile A. In contrast, nurses with lower degrees were most often represented in profile C, and these differences were statistically significant (*χ* ^2^ = 36.923, *p* < 0.001). The general work experience (in years) did not significantly differ among the clusters.

The nursing students' subsample did not show statistically significant differences regarding the course attended nor the year of study between the three clusters (*χ* ^2^ = 2.963, *p* = 0.227 and *χ* ^2^ = 9.047, *p* = 0.060, respectively). Table 2 reports the key characteristics of the profiles in the overall sample and among nurses and nursing students.

**TABLE 2:** Sample (*n* = 1756) characteristics based on their distribution to profiles A, B, and C.

Characteristics	Profile A (*n* = 678)	Profile B (*n* = 732)	Profile C (*n* = 346)	(*F* ^a^/*χ* ^2^)^b^	*p*‐value
Age in years, Mean (SD)	36.75 (14.25)	33.67 (12.97)	33.50 (11.92)	*F* = 11.62	**<0.001** *
Gender, *n* (%)					
Female	555 (81.9)	612 (83.6)	301 (87.0)	*χ* ^2^ = 4.162	0.384
Male	108 (15.9)	107 (14.6)	41 (11.8)		
Missing values	15 (2.2)	13 (1.8)	4 (1.2)		
Role, *n* (%)					
Nurses	383 (56.5)	329 (44.9)	147 (42.5)	*χ* ^2^ = 25.908	**<0.001**
Nursing students	295 (43.5)	403 (55.1)	199 (57.5)		
Country, *n* (%)					
United Kingdom	260 (38.3)	193 (26.4)	121 (35.0)	*χ* ^2^ = 45.974	**<0.001**
Finland	244 (36.0)	232 (31.7)	109 (31.5)		
Italy	174 (25.7)	307 (41.9)	116 (33.5)		
Social Media Apps, Mean (SD)	(*n* = 562)	(*n* = 577)	(*n* = 255)		
Facebook	2.54 (1.09)	2.59 (1.02)	2.62 (1.01)	*F* = 0.598	0.550
YouTube	2.48 (0.81)	2.39 (0.78)	2.42 (0.81)	*F* = 2.008	0.135
WhatsApp	3.57 (0.70)	3.62 (0.65)	3.58 (0.68)	*F* = 0.855	0.426
Instagram	2.86 (1.13)	3.07 (1.05)	2.95 (1.09)	*F* = 5.505	**0.004** **
TikTok	1.99 (1.22)	2.19 (1.26)	2.24 (1.23)	*F* = 5.587	**0.004** *
Snapchat	1.58 (1.05)	1.69 (1.13)	1.85 (1.17)	*F* = 5.268	**0.005** *
Pinterest	1.57 (0.72)	1.58 (0.70)	1.45 (0.70)	*F* = 3.192	**0.041** ***
Reddit	1.25 (0.66)	1.08 (0.31)	1.09 (0.33)	*F* = 18.928	**<0.001** *
LinkedIn	1.40 (0.71)	1.26 (0.55)	1.16 (0.42)	*F* = 15.810	**<0.001** *
Twitter/X	1.80 (1.07)	1.51 (0.82)	1.31 (0.69)	*F* = 29.422	**<0.001** *
Bergen Social Media Addiction Scale, mean (SD)	(*n* = 560) 1.99 (0.88)	(*n* = 575) 1.97(0.96)	(*n* = 251) 2.04 (0.96)	*F* = 0.594	0.552
Get vaccinated for influenza, *n* (%)	(*n* = 678)	(*n* = 732)	(*n* = 346)		
Yes	522 (77.0)	432 (59.0)	120 (34.7)	*χ* ^2^ = 198.874	**<0.001**
No	77 (11.4)	167 (22.8)	158 (45.7)		
Not every year	79 (11.7)	133 (18.2)	68 (19.7)		
Been affected by COVID‐19, *n* (%)	(*n* = 678)	(*n* = 732)	(*n* = 346)		
No	114 (16.8)	156 (21.2)	59 (17.1)	*χ* ^2^ = 10.446	0.107
Once	374 (55.2)	387 (52.9)	175 (50.6)		
Twice	140 (20.6)	134 (18.3)	75 (21.7)		
More than twice	50 (7.4)	55 (7.5)	37 (10.7)		
Vaccinated against COVID‐19, *n* (%)	(*n* = 678)	(*n* = 732)	(*n* = 346)		
Yes	660 (97.3)	687 (93.9)	259 (74.9)	*χ* ^2^ = 157.528	**<0.001**
No	18 (2.7)	45 (6.1)	87 (25.1)		
Nurses only					
Nursing role, *n* (%)	(*n* = 358)	(*n* = 323)	(*n* = 146)	*χ* ^2^ = 15.716	**0.003**
Clinical nurse	231 (64.5)	230 (71.2)	112 (76.7)		
Nurse leader	100 (27.9)	58 (18.0)	26 (17.8)		
Nurse educator	27 (7.5)	35 (10.8)	8 (5.5)		
Areas of practice, *n* (%)	(*n* = 383)	(*n* = 328)	(*n* = 147)		
Community	77 (20.1)	39 (11.9)	25 (17.0)	*χ* ^2^ = 8.723	**0.013**
Hospital settings	306 (79.9)	289 (88.1)	122 (83.1)		
Work experience in years, Mean (SD)	(*n* = 383) 9.86 (9.40)	(*n* = 329) 10.24 (9.52)	(*n* = 147) 8.47 (8.93)	*F* = 1.852	0.158
Worked in nursing (years post qualification), Mean (SD)	(*n* = 383) 20.31 (12.73)	(*n* = 329) 17.82 (11.61)	(*n* = 147) 16.21 (12.45)	*F* = 7.195	**<0.001** *
Highest academic award, *n* (%)	(*n* = 383)	(*n* = 328)	(*n* = 147)	*χ* ^2^ = 36.921	**<0.001**
Diploma/Advanced Diploma	82 (21.4)	98 (29.8)	54 (36.7)		
BSc/BSc (Hon)	173 (45.2)	173 (52.6)	71 (48.3)		
MSc/PhD	128 (33.4)	57 (17.6)	22 (15.0)		
Nursing students only					
Level of study, *n* (%)	(*n* = 295)	(*n* = 403)	(*n* = 199)	*χ* ^2^ = 2.963	0.227
Undergraduate	288 (97.6)	386 (95.8)	195 (97.9)		
Postgraduate	7 (2.4)	17 (4.2)	4 (2.1)		
Year of study (undergraduate)	(*n* = 288)	(*n* = 386)	(*n* = 195)	*χ* ^2^ = 9.047	0.060
First year	105 (36.5)	139 (36.0)	56 (28.8)		
Second year	96 (33.3)	148 (38.3)	90 (46.1)		
Third year	87 (30.2)	99 (25.7)	49 (25.1)		

*Note*: The mean difference is statistically significant at *p* < 0.05 (highlighted in bold). Percentages may not add to 100% due to rounding.

* Clusters A and C differed significantly in age (*p* < 0.001), working in nursing (*p* < 0.001), using TikTok (*p* < 0.01), Snapchat (*p* < 0.01), Reddit (*p* < 0.001), LinkedIn (*p* < 0.001), and Twitter (*p* < 0.001) variables based on the one‐way ANOVA *F* test, including multiple comparisons with Bonferroni correction.

** Clusters A and B differed significantly in using Instagram (*p* < 0.01) variable based on the one‐way ANOVA *F* test, including multiple comparisons with Bonferroni correction.

*** Clusters B and C differed significantly in using Pinterest (*p* < 0.05) variable based on the one‐way ANOVA *F* test, including multiple comparisons with Bonferroni correction.

^a^ One‐way ANOVA *F* test, including multiple pairwise comparisons conducted with Bonferroni correction.

^b^ Chi‐squared test and Fisher's exact test performed if the expected frequency of cells was <20%.

Nurses and nursing students significantly differed regarding social media use and platforms. In detail, nursing students showed higher scores in the BSMAS, lower frequency in the use of Facebook, LinkedIn, and Twitter/X, and higher scores in the use of YouTube, Instagram, TikTok, Snapchat, Pinterest, and Reddit. A detailed overview is reported in Table S1.

## DISCUSSION

This study aimed to identify patterns of characteristics and attitudes that contributed to vaccine hesitancy toward the COVID‐19 and influenza vaccines in an international sample of nurses and nursing students. As such, three distinct profiles were identified. Profile A displayed low hesitancy, profile B displayed slight hesitancy, and profile C displayed the highest levels of hesitancy toward COVID‐19 and influenza vaccines. The three profiles identified in this study are consistent with previous research, which has termed such degrees of hesitancy as vaccine‐confident (profile A), vaccine‐skeptical/hesitant (profile B), and vaccine‐refusal (profile C) (Heyerdahl et al., 2023). Similar research involving Italian HCWs identified four distinct profiles, which the authors termed the vaccine‐believer (low hesitancy), middle (average levels of hesitancy), hesitant (higher levels of hesitancy), and rejector (extreme levels of hesitancy) (Portoghese et al., 2023). Other studies reported five (Leung et al., 2022) and eight distinct profiles of hesitancy (Howard, 2023), but this study did not find evidence to support more than three distinct profiles. These previous studies have profiled participants using the 3C model of vaccine hesitancy (complacency, convenience, and confidence) proposed by the WHO SAGE Working Group on Vaccine Hesitancy (MacDonald, 2015), while others have used adapted versions such as the 5C model (adapted to include calculation and collective responsibility) (Betsch et al., 2018; MacDonald, 2015). However, there is an argument that studies should move beyond merely assessing behavioral barriers and consider additional characteristics when profiling populations for vaccine hesitancy (Howard, 2023). As such, this study discerned particular attitudes and characteristics that contribute to the three distinct profiles identified within a sample of nurses and nursing students, and hence, it moved beyond the traditional models by disclosing further characteristics of vaccine hesitancy.

The findings of this study suggest that various sociodemographic and occupational factors, such as age and, when considering nurses, academic qualifications, professional work experience, and roles in the nursing profession, are all associated with different levels of vaccine hesitancy. In particular, younger nurses with less professional experience in nursing and nursing students were more likely to be clustered in profiles B and C, indicating higher hesitancy toward vaccination. On the contrary, older nurses with more professional and educational experience, particularly those in senior roles such as nurse leaders and educators, exhibited lower hesitancy and were more inclined to accept vaccination (profile A). Previous research exploring associations between age and vaccine hesitancy yields inconclusive results. Some studies report that older nurses are more vaccine‐hesitant than younger nurses (Tomietto et al., 2022), while other studies align with our finding that older nurses are more accepting of a vaccine than younger nurses (Khubchandani et al., 2022). Although age significantly differed between the clusters identified in this study, the mean age difference between each profile is relatively small (3‐year mean difference between profile A and profile C). Therefore, we cannot draw any concrete inferences regarding targeting specific age groups for intervention. Instead, focusing on educational and occupational factors is more insightful. In addition to our evidence, previous research has also found vaccine hesitancy to be lower among nurses in higher roles (e.g., nursing supervisors) compared to entry‐level nursing roles (e.g., preregistered nurses and nursing students) (Manning et al., 2021). Similarly, nurses with lower educational attainment were also more hesitant to accept a COVID‐19 vaccine (Pataka et al., 2021), which was also found in this study as nurses with lower educational attainment were predominately clustered in profile C (higher rates of vaccine hesitancy). This evidence suggests that intervention campaigns to reduce vaccine hesitancy should prioritize targeting nurses in lower occupational roles and those with lower educational attainment.

In addition, the participants' countries of employment were also significantly different across the three profiles. In detail, British participants were predominantly associated with lower hesitancy (profile A). In contrast, Italian participants clustered more into profiles B and C, indicating higher levels of hesitancy and refusal toward vaccines, respectively. Finnish participants were relatively evenly split among all three profiles, suggesting a varied range of attitudes toward vaccinations. Although individuals display such hesitancy toward vaccinations, there was a high uptake of the COVID‐19 vaccine in each profile (97%, 94%, and 75% of participants had been vaccinated against COVID‐19 in profiles A, B, and C, respectively). The high rates of uptake of the COVID‐19 vaccine in this sample may be more reflective of the public health policies adopted in the respective countries, particularly mandatory COVID‐19 vaccinations for healthcare workers (Peruch et al., 2022). While mandates might have been instrumental in improving COVID‐19 vaccine uptake among this population, our findings suggest that this may have been in contrast with individual attitudes and intentions. Consequently, for some nurses in this sample, vaccination mandates may have been considered unethical, potentially infringing on the individual's autonomy and rights to make their own health decisions (Karlsson et al., 2023; Maneze et al., 2023). This finding is particularly interesting when compared to vaccination uptake for the influenza vaccine, where we can see the impact of an individual's attitudes and beliefs. In this sample, 77% of individuals in profile A receive the influenza vaccine annually, contrasting with only 35% of individuals accepting the vaccination in profile C. Together, these findings highlight the importance of building overall confidence in vaccines at the individual level. Intervention strategies should focus on increasing confidence in vaccines in general, as this will help an individual adopt more vaccine‐positive behaviors that would be utilized throughout their career and lifespan (as demonstrated in profile A).

This study also explored nurses' attitudes toward vaccinations. Overall, attitudes within each profile remained relatively consistent regardless of the vaccine under consideration. For instance, nurses in profile C exhibited heightened concerns about unforeseen future effects, mistrust in vaccine benefits, and worries about commercial profiteering for both COVID‐19 and influenza vaccines. In contrast, nurses in profile A displayed a high level of confidence in both vaccines and, mostly, did not share the same attitudes as nurses in profile C. The only exception was that nurses in profile A expressed slight concerns about the unforeseen future effects of both vaccines; however, these concerns did not seem to hinder their acceptance of either vaccine. Attitudes expressed by nurses in profile B were slightly greater than in profile A but were not as extreme as those by nurses in profile C. Our findings are supported by previous research that has also found that these specific attitudes are significantly associated with higher levels of vaccine hesitancy among nurses (Gallant et al., 2021; Tomietto et al., 2022). In addition, lack of trust in the government or the healthcare system (Nomura et al., 2021) and concerns about vaccine safety due to rapid development and expedition processes (Wise, 2021) have also contributed to vaccine hesitancy in this population. One main concern from our findings was that the unforeseen effects of vaccines were a consistent worry for participants across all three profiles. This attitude is of concern as it has been shown to increase an individual's perceptions of risk, leading them to seek information from other sources to inform their vaccination behavior (Dubé et al., 2021).

This is problematic as there is the risk of exposure to vaccine‐critical discourse or misinformation, which can increase postvaccination anxieties, undermine trust in institutions and governments, and exacerbate hesitant attitudes, ultimately hindering uptake (Yaqub et al., 2014). As such, intervention campaigns must address vaccine safety and side effects concerns. However, such strategies must avoid providing contradictory information over time, as such inconsistencies could be used to support vaccine‐critical discourses, thus undermining the effectiveness of the intervention. In particular, exposure to vaccine‐critical content or misinformation on the Internet has been shown to increase negative attitudes toward vaccination (Cascini et al., 2022). This was highly apparent throughout the COVID‐19 pandemic, where disinformation and misrepresentations about the vaccine were widely circulated on social media (WHO, 2021). In addition, the social media platform that the individual utilizes regularly also plays an instrumental role in shaping vaccination attitudes and subsequent behavior: Frequent users of Instagram, YouTube, Snapchat, and TikTok were more likely to be unwilling to accept a COVID‐19 vaccine, while users of Facebook and X displayed higher levels of vaccine willingness (Jennings et al., 2021).

This study highlighted that individuals frequently using TikTok, Snapchat, or Instagram were significantly more hesitant to receive vaccines (profiles B and C) than individuals with less engagement with these platforms (profile A). On the other hand, vaccine‐confident individuals (profile A) show more engagement with Reddit, LinkedIn, and X. In contrast to Jennings et al. (2021), we did not find significant differences in vaccine hesitancy based on engagement with Facebook or YouTube. Such reasons for the link between social media and vaccine hesitancy may be partly due to the algorithms employed by social media platforms. These algorithms tailor content based on the user's engagement history, meaning that individuals may find themselves in an echo chamber where they only see vaccine‐critical content or misinformation (Cascini et al., 2022; Rathje et al., 2022). Furthermore, individuals can find it hard to distinguish valid and relevant information from false or misleading information (Lee et al., 2022). As such, intervention campaigns should collaborate with social media platforms to ensure that the information circulated on their platform is reliable. Additionally, further investment is necessary to refine platform algorithms to ensure a balanced presentation of information from credible sources to prevent prolonged exposure in negative echo chambers. Moreover, educational campaigns are needed to help nurses identify reliable and trustworthy sources and critically analyze information. The findings of this study also indicate that using specific social media platforms (e.g., Instagram and TikTok) to disseminate vaccination campaigns may help to reach the most hesitant audiences of nurses and nursing students. In particular, nursing students significantly engage more than nurses with social media and they are frequent users of social media platforms linked to the most hesitant cluster.

Overall, this study has identified several avenues for intervention to reduce vaccine hesitancy in the nursing community. In particular, a multifaceted intervention that targets individual, community, and societal factors may be the most effective approach. At the individual level, the evidence from this study would suggest that vaccination campaigns should be tailored toward nursing students and nurses in entry‐level roles. One potential way would be to provide an in‐depth education package on vaccination as part of nursing undergraduate education. This would educate nursing students on the development, importance, and safety of vaccines and how to critically analyze vaccine‐related information, identify mis/disinformation on social media, and find information from trustworthy sources. As observed in nurses within profile A, maintaining vaccine confidence resulted in a consistent uptake of vaccinations. Therefore, fostering these positive attitudes and behaviors in early career nurses is crucial for establishing lifelong vaccine uptake.

At the community level, workplaces and educational institutions should establish confidential and informal spaces for nursing students and entry‐level nurses to discuss vaccination concerns with a trusted senior nurse. These conversations must focus on informational needs rather than forcing an individual toward vaccination. This strategy would help ensure that the individual's concerns and questions are answered and supported by a vaccine‐confident colleague or senior. In addition, having a positive role model will help promote vaccine confidence and reduce vaccine hesitancy. At the societal level, intervention strategies to address vaccine hesitancy in nurses could involve targeted campaigns on social media platforms: Focusing on platforms such as Instagram and TikTok may help to reach the most hesitant audiences of nurses and nursing students. Such campaigns should provide accurate and accessible information about vaccine safety, efficacy, and benefits to help address negative attitudes and promote vaccine uptake in this population. In addition, collaborating with trusted figures within the nursing community to share vaccine‐positive messages on these social media platforms could enhance the effectiveness of the intervention.

## LIMITATIONS

This study has several limitations. First, this study is limited in generalizability as the sample was predominately female. It may be that the characteristics identified for each profile may differ for male populations, as research indicates that males are generally less hesitant to accept vaccinations than females (Nery Jr et al., 2022). Similarly, although we captured a diverse age range (18–70 years) of participants, the mean ages of all three profiles were similar (33–36 years), which limited our ability to identify specific age groups associated with varying levels of hesitancy. We also did not capture any data pertaining to ethnicity, which is another key demographic variable associated with vaccine hesitancy, of which the factors and reasons for such hesitancy also vary between ethnic groups. Future research should include a comprehensive range of ethnicities, ages, and genders to ensure a more representative understanding of vaccination attitudes and behaviors across diverse populations and workforces. Another limitation of this study is the exclusive focus on Western countries. As such, this restricts the applicability of the findings to nurses in non‐Western countries. Previous research has used the 3C and 5C models of vaccine hesitancy (MacDonald, 2015), which include some of the factors mentioned above and have found differences in the number of clusters and the respective characteristics identified (Leung et al., 2022; Portoghese et al., 2023). As such, there is a gap in the literature for studies that utilize both measures of vaccine hesitancy (i.e., the 5C model and the VAX scale) to determine a more comprehensive profiling assessment of hesitancy in nurses and nursing students. Moreover, nurses and nursing students showed a significant difference in social media use. Future research should explore separately the nurses and the nursing students' clusters regarding social media to further tailor policy recommendations.

## CONCLUSIONS

This study profiled nurses and nursing students from Italy, Finland, and the United Kingdom to determine patterns of sociodemographic and occupational characteristics and attitudes toward vaccines (COVID‐19 and influenza) that contribute to vaccine hesitancy. Three profiles of hesitancy were identified, which consisted of clusters of individuals who were vaccine‐confident, vaccine‐hesitant, and vaccine‐refusers. Each profile exhibited specific characteristics, which provided insights into the demographics of hesitant individuals, the attitudes requiring intervention, and the platforms that can be utilized to reach the target population. Recommendations for global vaccination campaigns include utilizing educational and communication resources to inform, support, and address concerns among nursing students and early‐entry nurses. This strategy will help foster positive attitudes and behaviors toward vaccinations, ensuring a consistent uptake of vaccinations throughout the individual's nursing career and beyond.

## CLINICAL RESOURCES

Nursing Impact Project. (2023). Implementing Profiling to Boost Vaccination Uptake Among Nurses and Nursing Students. https://nursingimpactproject.wordpress.com/.

Unsworth, J., McCready, J., Erfani, G., Nichol, B., Gordon, C., Tomietto, M. (2023). Policy document: Vaccination and the registered nursing workforce. Preprint on OSF10.17605/OSF.IO/X3U95.

World Health Organization. (2015). Summary WHO SAGE conclusions and recommendations on vaccine hesitancy. https://www.who.int/docs/default‐source/immunization/demand/summary‐of‐sage‐vaccinehesitancy‐en.pdf?sfvrsn=abbfd5c8_2f‐sage‐vaccinehesitancy‐en.pdf.

## FUNDING INFORMATION

This study was funded by Sigma International—Sigma Global Nursing Research Grant and Northumbria University.

## CONFLICT OF INTEREST STATEMENT

The authors have no conflicts of interest to declare.

## Supporting information


Table S1.

## Data Availability

The data that support the findings of this study are available on request from the corresponding author. The data are not publicly available due to privacy or ethical restrictions.

## APPENDIX A

The Sigma IMPACT Research team collaborators:

Charlotte Gordon, MSc, PGCE, FHEA, Adv. Dip (Nurs), RN, BSc (Hons), Assistant Professor, Department of Nursing, Midwifery and Health, Faculty of Health and Life Sciences, Northumbria University, Newcastle upon Tyne, United Kingdom; email: charlotte.gordon@northumbria.ac.uk; ORCID: https://orcid.org/0000‐0002‐1659‐155X; John Unsworth, PhD, LLM, MSc, BSc (Hons), BA, RN, PGCE, NTF, PFHEA, FEANS, FFNMRCSI, Professor, Department of Nursing, Midwifery and Health, Faculty of Health and Life Sciences, Northumbria University, Newcastle upon Tyne, United Kingdom. john.unsworth@northumbria.ac.uk; ORCID: https://orcid.org/0000‐0002‐4150‐6513; Michelle Croston, PhD, RN. Manchester University NHS Foundation Trust. michelle.croston@mft.nhs.uk; ORCID: https://orcid.org/0000‐0002‐5479‐9853; Valentina Simonetti, PhD, MSc, RN, Associate Professor, Department of Innovative Technologies in Medicine and Dentistry, “Gabriele D'Annunzio” University of Chieti, 66100 Chieti, Italy; v.simonetti@unich.it. ORCID: https://orcid.org/0000‐0002‐7185‐4850; Bethany Nichol, MSc, Research Assistant, Department of Social Work, Education and Community Wellbeing, Faculty of Health and Life Sciences, Northumbria University, Newcastle upon Tyne, United Kingdom; bethany.nichol@northumbria.ac.uk; ORCID: https://orcid.org/0000‐0002‐7642‐1448.

## References

[jnu13016-bib-0001] Andreassen, C. S. , Pallesen, S. , & Griffiths, M. D. (2017). The relationship between addictive use of social media, narcissism, and self‐esteem: Findings from a large national survey. Addictive Behaviors, 64, 287–293. 10.1016/j.addbeh.2016.03.006 27072491

[jnu13016-bib-0002] Asad, H. , Johnston, C. , Blyth, I. , Holborow, A. , Bone, A. , Porter, L. , Tidswell, P. , & Healy, B. (2020). Health care workers and patients as Trojan horses: A COVID19 ward outbreak. Infection Prevention in Practice, 2(3), 100073. 10.1016/j.infpip.2020.100073 34316562 PMC7334135

[jnu13016-bib-0003] Betsch, C. , Schmid, P. , Heinemeier, D. , Korn, L. , Holtmann, C. , & Böhm, R. (2018). Beyond confidence: Development of a measure assessing the 5C psychological antecedents of vaccination. PLoS One, 13(12), e0208601. 10.1371/journal.pone.0208601 30532274 PMC6285469

[jnu13016-bib-0004] Cascini, F. , Pantovic, A. , Al‐Ajlouni, Y. A. , Failla, G. , Puleo, V. , Melnyk, A. , Lontano, A. , & Ricciardi, W. (2022). Social media and attitudes towards a COVID‐19 vaccination: A systematic review of the literature. EClinicalMedicine, 48, 101454. 10.1016/j.eclinm.2022.101454 35611343 PMC9120591

[jnu13016-bib-0056] Cornock, M. (2018). General Data Protection Regulation (GDPR) and implications for research. Maturitas, 111, A1–A2. 29395455 10.1016/j.maturitas.2018.01.017

[jnu13016-bib-0005] Data Protection Act . (2018). c.12. https://www.legislation.gov.uk/ukpga/2018/12/contents/enacted

[jnu13016-bib-0006] Department of Health and Social Care . (2018). Regulations making COVID‐19 vaccination a condition of deployment to end. https://www.gov.uk/government/news/regulations‐making‐covid‐19‐vaccination‐a‐condition‐of‐deployment‐to‐end

[jnu13016-bib-0007] DeVellis, R. F. , & Thorpe, C. T. (2021). Scale development: Theory and applications. Sage publications.

[jnu13016-bib-0008] Drew, L. (2019). The case for mandatory vaccination. Nature, 575(7784), S58–S60. 10.1038/d41586-019-03642-w 31776503

[jnu13016-bib-0009] Dubé, È. , Ward, J. K. , Verger, P. , & MacDonald, N. E. (2021). Vaccine hesitancy, acceptance, and anti‐vaccination: Trends and future prospects for public health. Annual Review of Public Health, 42(1), 175–191. 10.1146/annurev-publhealth-090419-102240 33798403

[jnu13016-bib-0010] EUR‐Lex . (2019). Directive 2013/55/EU of the European Parliament and of the council of 20 November 2013 amending directive 2005/36/EC on the recognition of professional qualifications and regulation (EU) No 1024/2012 on administrative cooperation through the internal market information system (‘the IMI Regulation’). https://eur‐lex.europa.eu/legal‐content/EN/ALL/?uri=celex%3A32013L0055

[jnu13016-bib-0011] European Center for Disease Control and Prevention [ECDC] . (2018). State Healthcare Worker and Patient Vaccination Laws. https://www.cdc.gov/phlp/publications/topic/vaccinationlaws.html

[jnu13016-bib-0013] Gallant, A. J. , Nicholls, L. A. B. , Rasmussen, S. , Cogan, N. , Young, D. , & Williams, L. (2021). Changes in attitudes to vaccination as a result of the COVID‐19 pandemic: A longitudinal study of older adults in the UK. PLoS One, 16(12), e0261844. 34941951 10.1371/journal.pone.0261844 PMC8699689

[jnu13016-bib-0014] Heyerdahl, L. W. , Dielen, S. , Dodion, H. , van Riet, C. , Nguyen, T. , Simas, C. , Boey, L. , Kattumana, T. , Vandaele, N. , Larson, H. J. , Grietens, K. P. , Giles‐Vernick, T. , & Gryseels, C. (2023). Strategic silences, eroded trust: The impact of divergent COVID‐19 vaccine sentiments on healthcare workers' relations with peers and patients. Vaccine, 41(4), 883–891. 36319488 10.1016/j.vaccine.2022.10.048 PMC9606030

[jnu13016-bib-0015] Howard, M. C. (2023). Integrating the person‐centered approach with the study of vaccine hesitancy: Applying latent profile analysis to identify vaccine hesitancy subpopulations and assess their relations with correlates and vaccination outcomes. Vaccine, 41(33), 4823–4835. doi:10.1016/j.vaccine.2023.06.057 37357075

[jnu13016-bib-0016] IBM Corp . (2021). IBM SPSS statistics for Windows, Version 28.0. IBM Corp.

[jnu13016-bib-0017] Ikotun, A. M. , Ezugwu, A. E. , Abualigah, L. , Abuhaija, B. , & Heming, J. (2023). K‐means clustering algorithms: A comprehensive review, variants analysis, and advances in the era of big data. Information Sciences, 622, 178–210. 10.1016/j.ins.2022.11.139

[jnu13016-bib-0018] Jennings, W. , Stoker, G. , Bunting, H. , Valgarðsson, V. O. , Gaskell, J. , Devine, D. , McKay, L. , & Mills, M. C. (2021). Lack of trust, conspiracy beliefs, and social media use predict COVID‐19 vaccine hesitancy. Vaccine, 9(6), 593. doi:10.3390/vaccines9060593 PMC8226842 34204971

[jnu13016-bib-0019] Karlsson, L. C. , Garrison, A. , Holford, D. , Fasce, A. , Lewandowsky, S. , Taubert, F. , Schmid, P. , Betsch, C. , Rodrigues, F. , Fressard, L. , Verger, P. , & Soveri, A. (2023). Healthcare professionals' attitudes to mandatory COVID‐19 vaccination: Cross‐sectional survey data from four European countries. Human Vaccines & Immunotherapeutics, 19(2), 2256442. 10.1080/21645515.2023.2256442 37724556 PMC10512846

[jnu13016-bib-0020] Khubchandani, J. , Bustos, E. , Chowdhury, S. , Biswas, N. , & Keller, T. (2022). COVID‐19 vaccine refusal among nurses worldwide: Review of trends and predictors. Vaccine, 10(2), 230. 10.3390/vaccines10020230 PMC8876951 35214687

[jnu13016-bib-0021] Kline, R. B. (2015). Principles and practice of structural equation modeling. Guilford Publications.

[jnu13016-bib-0022] Lazarus, J. V. , Wyka, K. , White, T. M. , Picchio, C. A. , Gostin, L. O. , Larson, H. J. , Rabin, K. , Ratzan, S. C. , Kamarulzaman, A. , & El‐Mohandes, A. (2023). A survey of COVID‐19 vaccine acceptance across 23 countries in 2022. Nature Medicine, 29(2), 366–375. 10.1038/s41591-022-02185-4 36624316

[jnu13016-bib-0023] Lee, S. K. , Sun, J. , Jang, S. , & Connelly, S. (2022). Misinformation of COVID‐19 vaccines and vaccine hesitancy. Scientific Reports, 12(1), 13681. 10.1038/s41598-022-17430-6 35953500 PMC9366757

[jnu13016-bib-0024] Leung, C. L. K. , Li, K. K. , Wei, V. W. I. , Tang, A. , Wong, S. Y. S. , Lee, S. S. , & Kwok, K. O. (2022). Profiling vaccine believers and skeptics in nurses: A latent profile analysis. International Journal of Nursing Studies, 126, 104142. 10.1016/j.ijnurstu.2021.104142 34923316 PMC8676577

[jnu13016-bib-0025] MacDonald, N. E. (2015). Vaccine hesitancy: Definition, scope and determinants. Vaccine, 33(34), 4161–4164. 10.1016/j.vaccine.2015.04.036 25896383

[jnu13016-bib-0026] Maltezou, H. C. , Dounias, G. , Rapisarda, V. , & Ledda, C. (2022). Vaccination policies for healthcare personnel: Current challenges and future perspectives. Vaccine, X, 11, 100172. 10.1016/j.jvacx.2022.100172 PMC9190304 35719325

[jnu13016-bib-0027] Maneesriwongul, W. , & Dixon, J. K. (2004). Instrument translation process: A methods review. Journal of Advanced Nursing, 48(2), 175–186. 10.1111/j.1365-2648.2004.03185.x 15369498

[jnu13016-bib-0028] Maneze, D. , Salamonson, Y. , Grollman, M. , Montayre, J. , & Ramjan, L. (2023). Mandatory COVID‐19 vaccination for healthcare workers: A discussion paper. International Journal of Nursing Studies, 138, 104389. 10.1016/j.ijnurstu.2022.104389 36462385 PMC9709452

[jnu13016-bib-0029] Manning, M. L. , Gerolamo, A. M. , Marino, M. A. , Hanson‐Zalot, M. E. , & Pogorzelska‐Maziarz, M. (2021). COVID‐19 vaccination readiness among nurse faculty and student nurses. Nursing Outlook, 69(4), 565–573. 10.1016/j.outlook.2021.01.019 33610324 PMC7862894

[jnu13016-bib-0030] Martin, L. R. , & Petrie, K. J. (2017). Understanding the dimensions of anti‐vaccination attitudes: The vaccination attitudes examination (VAX) scale. Annals of Behavioral Medicine, 51(5), 652–660. 10.1007/s12160-017-9888-y 28255934

[jnu13016-bib-0031] Mathieu, E. , Ritchie, H. , Ortiz‐Ospina, E. , Roser, M. , Hasell, J. , Appel, C. , Giattino, C. , & Rodés‐Guirao, L. (2021). A global database of COVID‐19 vaccinations. Nature Human Behaviour, 5(7), 947–953. 10.1038/s41562-021-01122-8 33972767

[jnu13016-bib-0032] McCready, J. L. , Nichol, B. , Steen, M. , Unsworth, J. , Comparcini, D. , & Tomietto, M. (2023). Understanding the barriers and facilitators of vaccine hesitancy towards the COVID‐19 vaccine in healthcare workers and healthcare students worldwide: An umbrella review. PLoS One, 18(4), e0280439. 10.1371/journal.pone.0280439 37043505 PMC10096263

[jnu13016-bib-0033] Mikkonen, K. , Tomietto, M. , & Watson, R. (2022). Instrument development and psychometric testing in nursing education research. Nurse Education Today, 119, 105603. 10.1016/j.nedt.2022.105603 36244252

[jnu13016-bib-0034] Nery, N., Jr. , Ticona, J. P. A. , Cardoso, C. W. , Prates, A. P. P. B. , Vieira, H. C. A. , Salvador de Almeida, A. , et al. (2022). COVID‐19 vaccine hesitancy and associated factors according to sex: A population‐based survey in Salvador, Brazil. PLoS One, 17(1), e0262649. 10.1371/journal.pone.0262649 35061811 PMC8782400

[jnu13016-bib-0035] Nomura, S. , Eguchi, A. , Yoneoka, D. , Kawashima, T. , Tanoue, Y. , Murakami, M. , Sakamoto, H. , Maruyama‐Sakurai, K. , Gilmour, S. , Shi, S. , Kunishima, H. , Kaneko, S. , Adachi, M. , Shimada, K. , Yamamoto, Y. , & Miyata, H. (2021). Reasons for being unsure or unwilling regarding intention to take COVID‐19 vaccine among Japanese people: A large cross‐sectional national survey. The Lancet Regional Health–Western Pacific, 14, 100223. 10.1016/j.lanwpc.2021.100223 34368797 PMC8324415

[jnu13016-bib-0036] Pataka, A. , Kotoulas, S. , Stefanidou, E. , Grigoriou, I. , Tzinas, A. , Tsiouprou, I. , Zarogoulidis, P. , Courcoutsakis, N. , & Argyropoulou, P. (2021). Acceptability of healthcare professionals to get vaccinated against COVID‐19 two weeks before initiation of national vaccination. Medicina, 57(6), 611. 10.3390/medicina57060611 34204614 PMC8231122

[jnu13016-bib-0037] Patelarou, E. , Galanis, P. , Mechili, E. A. , Argyriadi, A. , Argyriadis, A. , Asimakopoulou, E. , Brokaj, S. , Bucaj, J. , Carmona‐Torres, J. M. , Cobo‐Cuenca, A. I. , Doležel, J. , Finotto, S. , Jarošová, D. , Kalokairinou, A. , Mecugni, D. , Pulomenaj, V. , Saliaj, A. , Sopjani, I. , Zahaj, M. , & Patelarou, A. (2021). Factors influencing nursing students' intention to accept COVID‐19 vaccination: A pooled analysis of seven European countries. Nurse Education Today, 104, 105010. 10.1016/j.nedt.2021.105010 34126322 PMC8189729

[jnu13016-bib-0038] Peruch, M. , Toscani, P. , Grassi, N. , Zamagni, G. , Monasta, L. , Radaelli, D. , Livieri, T. , Manfredi, A. , & D'Errico, S. (2022). Did Italy really need compulsory vaccination against COVID‐19 for healthcare workers? Results of a survey in a centre for maternal and child health. Vaccine, 10(8), 1293. 10.3390/vaccines10081293 PMC9414650 36016179

[jnu13016-bib-0040] Portoghese, I. , Siddi, M. , Chessa, L. , Costanzo, G. , Garcia‐Larsen, V. , Perra, A. , Littera, R. , Sambugaro, G. , del Giacco, S. , Campagna, M. , & Firinu, D. (2023). COVID‐19 vaccine hesitancy among Italian healthcare workers: Latent profiles and their relationships to predictors and outcome. Vaccine, 11(2), 273. 10.3390/vaccines11020273 PMC9964484 36851151

[jnu13016-bib-0041] Rathje, S. , He, J. K. , Roozenbeek, J. , van Bavel, J. J. , & van der Linden, S. (2022). Social media behavior is associated with vaccine hesitancy. PNAS Nexus, 1(4), pgac207. 10.1093/pnasnexus/pgac207 36714849 PMC9802475

[jnu13016-bib-0042] Royal College of Nursing . (2022). RCN position on mandating vaccination for health and social care staff . https://www.rcn.org.uk/About‐us/Our‐Influencing‐work/Position‐statements/rcn‐position‐on‐vaccination

[jnu13016-bib-0043] Salem, S. , Miligi, E. , Alanazi, H. H. , Alanazi, N. A. , & Alanazi, A. A. (2019). Knowledge and limitations associated with the uptake of seasonal influenza vaccine among nursing students. International Journal of Novel Research in Healthcare and Nursing, 6(1), 471–479.

[jnu13016-bib-0044] StataCorp . (2013). Stata Statistical Software: Release 13. StataCorp LP.

[jnu13016-bib-0045] Tabachnick, B. G. , Fidell, L. S. , & Ullman, J. B. (2013). Using multivariate statistics (Vol. 6, pp. 497–516). Pearson.

[jnu13016-bib-0046] Tobin, A. (2021). No jab, no job: Mandating workplace COVID‐19 vaccinations in the current legal landscape. https://www.hopgoodganim.com.au/page/knowledge‐centre/blog/no‐jab‐no‐job‐covid‐workplace‐vaccine‐mandates‐2021

[jnu13016-bib-0047] Tomietto, M. , Simonetti, V. , Comparcini, D. , Stefanizzi, P. , & Cicolini, G. (2022). A large cross‐sectional survey of COVID‐19 vaccination willingness amongst healthcare students and professionals: Reveals generational patterns. Journal of Advanced Nursing, 78(9), 2894–2903. 10.1111/jan.15222 35301774 PMC9111790

[jnu13016-bib-0048] Vandenbroucke, J. P. , Elm, E. V. , Altman, D. G. , Gøtzsche, P. C. , Mulrow, C. D. , Pocock, S. J. , Poole, C. , Schlesselman, J. J. , Egger, M. , & Initiative, S. (2007). Strengthening the reporting of observational studies in epidemiology (STROBE): Explanation and elaboration. Annals of Internal Medicine, 147(8), W‐163. 10.7326/0003-4819-147-8-200710160-00010-w1 17938389

[jnu13016-bib-0049] Wise, J. (2021). Covid‐19: European countries suspend use of Oxford‐AstraZeneca vaccine after reports of blood clots. BMJ, 372, n699. 10.1136/bmj.n699 33707182

[jnu13016-bib-0050] World Health Organization . (2015). Vaccine hesitancy: A growing challenge for immunization programmes . https://www.who.int/news/item/18‐08‐2015‐vaccine‐hesitancy‐a‐growing‐challenge‐for‐immunization‐programmes

[jnu13016-bib-0051] World Health Organization . (2018). Immunization, Vaccines and Biologicals: Addressing Vaccine Hesitancy. https://www.who.int/immunization/programmes_systems/vaccine_hesitancy/en/

[jnu13016-bib-0052] World Health Organization . (2019). How to implement seasonal influenza vaccination of health workers . https://www.who.int/publications‐detail‐redirect/9789241515597

[jnu13016-bib-0053] World Health Organization . (2020). COVID‐19 response . https://apps.who.int/gb/ebwha/pdf_files/WHA73/A73_R1‐en.pdf

[jnu13016-bib-0054] World Health Organization . (2021). Fighting misinformation in the time of COVID‐19, one click at a time. https://www.who.int/news‐room/feature‐stories/detail/fighting‐misinformation‐in‐the‐time‐of‐covid‐19‐one‐click‐at‐a‐time

[jnu13016-bib-0055] Yaqub, O. , Castle‐Clarke, S. , Sevdalis, N. , & Chataway, J. (2014). Attitudes to vaccination: A critical review. Social Science & Medicine, 112, 1–11. 10.1016/j.socscimed.2014.04.018 24788111

