# Supplementary material for: Profiling vaccine hesitancy in nursing to tailor public healthcare policies: A cross‐sectional international study
Source: J Nurs Scholarsh. 2024 Aug 14;57(2):253–65. doi: 10.1111/jnu.13016 (PMC11931988; doi:10.1111/jnu.13016)
Supplement: Supplementary file 1 — Table S1. [file JNU-57-253-s001.docx]

| **Variables** | **Group** | **n*** | **mean** | **SD** | **t** | **p-value** |
| --- | --- | --- | --- | --- | --- | --- |
| Bergen Social Media Addiction Scale | Nurses | 665 | 1.81 | 0.85 | -6.806 | <.001 |
|  | Nursing students | 721 | 2.15 | 0.96 | -6.806 | <.001 |
| Facebook | Nurses | 669 | 2.82 | 1.03 | 8.571 | <.001 |
|  | Nursing students | 725 | 2.35 | 1.01 | 8.571 | <.001 |
| YouTube | Nurses | 668 | 2.33 | 0.73 | -4.671 | <.001 |
|  | Nursing students | 725 | 2.53 | 0.84 | -4.671 | <.001 |
| WhatsApp | Nurses | 667 | 3.63 | 0.63 | 1.595 | .111 |
|  | Nursing students | 726 | 3.57 | 0.71 | 1.595 | .111 |
| Instagram | Nurses | 668 | 2.63 | 1.13 | -11.601 | <.001 |
|  | Nursing students | 725 | 3.28 | 0.95 | -11.601 | <.001 |
| TikTok | Nurses | 666 | 1.52 | 0.88 | -19.539 | <.001 |
|  | Nursing students | 726 | 2.67 | 1.27 | -19.539 | <.001 |
| Snapchat | Nurses | 666 | 1.24 | 0.66 | -15.294 | <.001 |
|  | Nursing students | 726 | 2.08 | 1.29 | -15.294 | <.001 |
| Pinterest | Nurses | 666 | 1.48 | 0.68 | -3.433 | <.001 |
|  | Nursing students | 726 | 1.61 | 0.72 | -3.433 | <.001 |
| Reddit | Nurses | 665 | 1.10 | 0.42 | -3.661 | <.001 |
|  | Nursing students | 726 | 1.20 | 0.55 | -3.661 | <.001 |
| LinkedIn | Nurses | 667 | 1.45 | 0.73 | 9.695 | <.001 |
|  | Nursing students | 725 | 1.15 | 0.41 | 9.695 | <.001 |
| Twitter/X | Nurses | 668 | 1.75 | 1.04 | 6.415 | <.001 |
|  | Nursing students | 726 | 1.44 | 0.78 | 6.415 | <.001 |

Supplementary table 1. Social Media use: comparison between nurses and nursing students.

* n differs from the overall sample because of missing values regarding these variables of the survey
